# Transition Shock Among Chinese New Nurses: A Systematic Review and Meta‐Analysis

**DOI:** 10.1155/jonm/9306693

**Published:** 2026-01-14

**Authors:** Jiexuan Xu, Jianqin Huang, Yu Zhai, Murong Lu, Xuemei Liu, Hongjing Yu

**Affiliations:** ^1^ Nursing Administration Department, The Second Affiliated Hospital of Guangzhou Medical University, Guangzhou, China, gzhmc.edu.cn; ^2^ Intensive Care Unit, The Second Affiliated Hospital of Guangzhou Medical University, Guangzhou, Guangdong, China, gzhmc.edu.cn; ^3^ Department of Cardiovascular Medicine, The Second Affiliated Hospital of Guangzhou Medical University, Guangzhou, Guangdong, China, gzhmc.edu.cn

**Keywords:** current status, influencing factors, meta-analysis, new nurses, transition shock

## Abstract

**Aim:**

To investigate the current level and influencing factors of transition shock among new nurses in China.

**Background:**

New nurses in China face multiple transition shock, which make them prone to burnout and more likely to leave the profession. A systematic analysis of their current status and influencing factors is needed to enhance their occupational well‐being and stabilize the nursing workforce.

**Methods:**

Computer searches were conducted to retrieve the literature on the current status and factors affecting the impact of the transition of new nurses in China from Chinese databases including China National Knowledge Infrastructure (CNKI), Wanfang Database, and China Biomedical Literature Database (CBM), as well as international databases including PubMed, Embase, Scopus, and the Cumulative Index to Nursing and Allied Health Literature (CINAHL). Meta‐analysis was performed by using Stata 17.0 and RevMan 5.3 software.

**Results:**

A total of 30 articles involving 12,459 Chinese new nurses were included, and 20 influencing factors were extracted. Meta‐analysis results showed that the transition shock score was 93.08 (95% CI: 87.28–98.89). The influencing factors of transition shock among new nurses in China include both personal factors (female, nonlocal origin, college degree or below, disliking or feeling neutral toward their department, choosing the nursing profession due to nonpersonal interests, low professional identity, poor psychological resilience, infrequent feedback‐seeking behavior, and service length ≤ 6 months) and external factors (nonestablishment employment type, monthly income ≤ 5000 RMB, > 7 night shifts per month, lack of family support for nursing work, and low social support) (*p* < 0.05).

**Conclusion:**

The transition shock of new nurses in China is at a moderate‐to‐high level and is influenced by multiple personal and work‐related factors. Nursing managers should develop targeted interventions based on these influencing factors to alleviate transition shock, improve new nurses’ career satisfaction, and enhance the quality of nursing services.

## 1. Introduction

The demand for nurses is growing worldwide as the global economy and healthcare systems continue to evolve [1]. The World Health Organization has estimated that about 40% of the clinical nursing workforce will retire by 2023; to compensate for this loss, nearly 12.9 million new nurses will be needed to maintain a steady nursing workforce [2].

Nurse shortages therefore represent a major global health workforce challenge and pose a threat to the quality, safety, and continuity of patient care.

China is one of the most populous countries in the world with a large healthcare workforce [3]. Over the past decade, the number of nurses in China has increased by an average of 8% annually, with more than 300,000 new nurses entering the profession each year, according to the National Health Commission of the People’s Republic of China [4]. However, despite this rapid growth in absolute numbers, the supply of nurses remains insufficient to meet the rapidly increasing healthcare needs of the population, and many medical institutions continue to experience heavy nursing workloads [5]. Newly recruited nurses constitute an important source of workforce replenishment, and their professionalism, clinical competence, and psychological tolerance have a direct impact on the overall quality of nursing care and patient satisfaction; they are regarded as the “new force” of the nursing team [6, 7].

When individuals move from a familiar role to a new one, they may experience transition shock, a term that refers to feelings of disorientation, confusion, doubt, and uncertainty arising from changes in roles, relationships, knowledge, and responsibilities [8]. For new nurses, the shift from student to practicing nurse is a crucial yet challenging process for retention and is often described as complex, dynamic, and highly demanding [9]. Several studies have indicated that transition shock is one of the main contributors to the high turnover rate among new nurses in the early stages of their careers [10, 11]. Gan et al. [12] reported that the turnover rate of new nurses in China in 2017 was 4.87%, whereas it was only 0.74% among nurses with more than 10 years of experience, suggesting that new nurses are much more likely to leave the profession than their experienced counterparts. Frequent negative emotions such as anxiety, depression, and irritability further demonstrate the substantial impact of transition shock on the mental health of new nurses, and prolonged transition shock may erode their professional identity [13], threatening the continuity and stability of their career development. In the context of a persistent nurse shortage, such issues may significantly hinder the stabilization and sustainable growth of the nursing workforce in China.

The turnover of Chinese new nurses, driven by transition‐related and psychological problems, leads to instability in the nursing team and may adversely affect the quality of nursing services, patient satisfaction, and overall hospital care. Therefore, focusing on Chinese new nurses is essential for understanding context‐specific determinants of transition shock and for developing targeted interventions to support their successful transition and retention. Although a number of Chinese studies have examined the current status and influencing factors of transition shock among new nurses, most are single‐center studies with limited sample sizes, and their findings are not entirely consistent. To provide an evidence‐based basis for preventive and targeted interventions, help new nurses navigate the transition period smoothly, and promote the long‐term and healthy development of the nursing and healthcare system, the present study used a meta‐analysis to systematically evaluate the current status and influencing factors of transition shock among new nurses in China.

## 2. Methods

The Cochrane Collaboration criteria were followed in the conduct of this study, and the Preferred Reporting Items for Systematic Reviews and Meta‐Analysis (PRISMA) statement was followed in its reporting. This study was prospectively registered in the PROSPERO database (registration number CRD42025639207).

### 2.1. Study Selection

#### 2.1.1. Inclusion Criteria

The inclusion criteria were as follows: (1) the study subjects were Chinese nurses who had been employed for less than 2 years and had obtained the professional qualification certificate for nurses [14]; (2) the study content was the current status of transition shock for new nurses and its influencing factors; (3) the outcome indicator was the total score on the Self‐Assessment Scale of Transition Shock for New Nurses, a standardized instrument specifically developed to assess transition shock among newly graduated nurses and widely used in Chinese studies; and (4) the literature was written in Chinese or English. As almost all eligible studies on Chinese new nurses adopted this instrument and reported its total score, using this score as the outcome indicator ensured that transition shock was measured in a consistent and comparable way across studies and reduced measurement heterogeneity related to different scales.

#### 2.1.2. Exclusion Criteria

The exclusion criteria were as follows: (1) reviews, conference abstracts, case reports, and duplicate publications; (2) literature from which valid data could not be extracted; and (3) low‐quality literature.

### 2.2. Literature Search

Using a computerized search, pertinent literature was retrieved from Chinese databases including China National Knowledge Infrastructure (CNKI), Wanfang Database, and the China Biomedical Literature Database (CBM), as well as international databases including PubMed, Embase, Scopus, and the Cumulative Index to Nursing and Allied Health Literature (CINAHL). The relevant literature was then added in a snowballing fashion, with the literature search covering all studies published up to December 2, 2024. MeSH and free‐text terms, such as “new nurs∗,” “novice nurs∗,” “newly graduated nurs∗,” “junior nurs∗,” “transition,” and “transition shock,” were combined to create the search terms.

### 2.3. Literature Selection and Data Extraction

In rigorous adherence to the inclusion and exclusion criteria, two meta‐learning researchers independently conducted literature screening and data extraction, including authors, year of publication, sample size, outcome metrics, and specific values. The final results were cross‐checked. Disagreements were discussed, and if required, a third researcher was consulted.

### 2.4. Assessment of Literature Quality

Two researchers independently assessed the included literature’s quality using the Agency for Healthcare Research and Quality’s (AHRQ) cross‐sectional study evaluation criteria [15], which consists of a total of 11 entries. High quality was defined as a total score of ≥ 8, medium quality as a score of 4–7, and low quality as a score of 0–3. Inconsistent evaluation results were discussed, and if required, a third researcher was consulted.

### 2.5. Statistical Methods

Statistical analyses were performed using RevMan 5.3 (Cochrane Collaboration, Oxford, UK) and Stata 17.0 (StataCorp LP, College Station, TX, USA). A fixed‐effects model was employed if *I* ^2^ ≤ 50% and *p* ≥ 0.1, indicating acceptable heterogeneity between studies; a random‐effects model was selected if *I* ^2^ > 50% and *p* < 0.1, indicating significant heterogeneity between studies. For continuous variables, the combined effect sizes were calculated using the standardized mean difference (SMD) and 95% confidence interval (CI); for the correlation coefficient *r*, the combined effect sizes were calculated using Summary Fisher’s *Z* and 95% CI. Differences were considered statistically significant at *p* < 0.05.

## 3. Results

### 3.1. Literature Search Results

**Figure 1:**
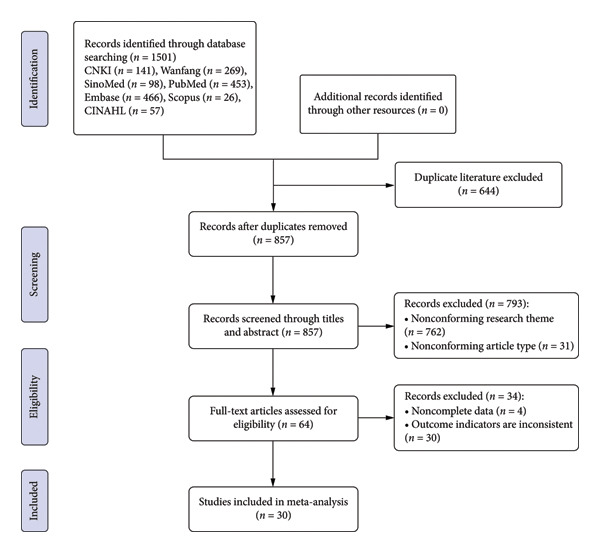
Literature screening process and results.

The initial search yielded 1501 relevant articles, and 857 relevant articles were obtained after removing duplicate publications by EndNote literature management software. After reviewing the abstracts and titles, 793 articles were disqualified. Thirty‐four articles that did not fit the inclusion criteria were eliminated after the full text was read in more detail. Ultimately, the meta‐analysis contained 30 articles. The literature screening process is illustrated in Figure 1.

### 3.2. Basic Characteristics and Quality Evaluation of the Included Studies

The meta‐analysis included 30 articles published from 2015 to 2025, including 29 papers in Chinese and 1 paper in English. The study sample totaled 12,459 cases. The literature was investigated using the new Nurse Transition Shock Self‐Rating Scale developed by Xue et al. [16]. The 30 articles had quality assessment scores ranging from 6 to 9, all of which fell into the medium‐ to high‐grade range. The specific results are shown in Table 1. The detailed AHRQ cross‐sectional study quality assessment checklist and scoring criteria are provided in Supporting Tables S1–S3.

**Table 1:** Basic characteristics and quality evaluation of the included studies.

Study	Sample size	Risk factors	Transition shock score	Quality
[17]	497	②⑤⑧⑨⑫	113.63 ± 8.24	7
[18]	133	①④⑥⑧⑨⑭⑯	91.50 ± 7.38	8
[19]	121	②③⑤⑥⑧⑰	96.44 ± 13.19	8
[20]	196	②③④⑤⑨⑩⑬	85.75 ± 16.38	7
[21]	400	①③⑤⑧⑨⑯	102.03 ± 8.55	6
[22]	416	①②③④⑤⑥⑦⑧⑨⑩⑪⑬⑮⑯⑰	87.05 ± 19.18	8
[23]	313	①④⑥⑧⑨⑩⑭⑲	88.87 ± 27.33	7
[24]	121	①③④⑥⑧⑩⑪⑫⑬	99.98 ± 19.16	9
[25]	230	④⑨⑩⑯	93.70 ± 16.57	9
[26]	236	①②④⑤⑦⑧⑩⑯	95.87 ± 16.45	7
[27]	824	①②⑧	126.80 ± 7.48	6
[28]	260	③⑧⑨	111.46 ± 12.34	8
[29]	70	①②③④⑧⑨⑩⑯	97.31 ± 2.65	6
[11]	1128	⑧⑨	89.93 ± 24.76	7
[30]	186	②④⑧⑨⑩⑭⑯⑰	110.36 ± 13.62	7
[31]	647	①②④⑤⑦⑧⑨⑩⑪⑫⑬⑮⑯	82.77 ± 23.33	8
[32]	200	③④⑤⑨⑩⑪⑬⑮⑯	82.88 ± 18.96	7
[33]	42	③⑨	109.45 ± 7.14	6
[34]	3342	①③④⑤⑥⑧⑨⑩⑫⑯	84.53 ± 22.52	8
[35]	227	①②③④⑤⑧⑨⑩⑬⑯⑰	95.2 ± 15.16	6
[36]	440	⑨⑰	86.98 ± 24.83	8
[37]	327	⑱	86.57 ± 25.15	7
[38]	314	⑱	84.03 ± 22.42	8
[39]	189	①⑩⑱	103.73 ± 6.26	7
[40]	363	⑲	78.98 ± 21.16	7
[41]	99	⑨⑲	72.01 ± 20.28	8
[42]	102	①④⑧⑩⑯⑲	89.8 ± 14.49	6
[43]	216	⑲	84.56 ± 12.66	9
[44]	299	③⑯⑱⑳	73.44 ± 20.52	9
[45]	521	⑳	86.35 ± 17.45	8

*Note:* ① age; ② service length (months), ③ place of origin, ④ only child status, ⑤ monthly income, ⑥ Frequency of night shifts, ⑦ weekly working hours, ⑧ education background, ⑨ gender, ⑩ employment type, ⑪ department liking, ⑫ hospital level, ⑬ family support, ⑭ registered residence, ⑮ reasons for choosing nursing profession, ⑯ marital status, ⑰ professional identity, ⑱ psychological resilience, ⑲ feedback‐seeking behavior, and ⑳ social support.

### 3.3. Meta‐Analysis Results on the Current Status of Transition Shock Among New Nurses in China

**Figure 2:**
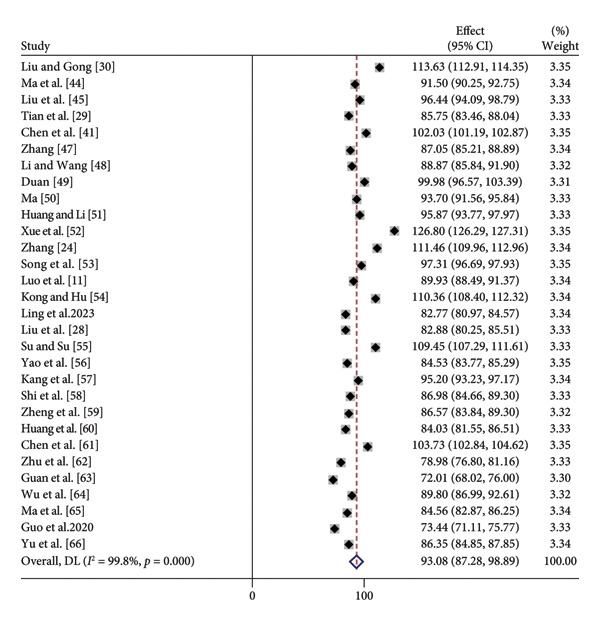
Meta‐analysis results on the current status of transition shock among new nurses in China.

All 30 articles reported on the current status of transition shock among new nurses in China, and a heterogeneity test of the included studies (*I* ^2^ = 99.8%, *p* < 0.01) indicated a high degree of heterogeneity among the included studies. No single study was shown to have a substantial influence on the overall findings using sensitivity analysis employing the stepwise removal technique. Consequently, the analysis was conducted using a random‐effects model, which showed that the transition shock score of new nurses in China was 93.08 (95% CI: 87.28–98.89, *p* < 0.01), as shown in Figure 2. In view of the very high heterogeneity, we further performed subgroup analyses by year of publication, length of service, and working department to explore potential sources of variability (Table 2).

**Table 2:** Subgroup analysis of the current status of transition shock among new nurses in China.

Subgroups	Number of included studies	Heterogeneity		Meta‐analysis result		
		*I* ^2^ (%)	*p*	SMD (95% CI)	*Z*	*p*
Year						
2015–2018	6	58	0.03	104.28	90.02–118.54	< 0.001
2019–2022	14	0	0.46	98.32	94.34–102.31	< 0.001
2023–2025	10	0	0.89	89.37	77.07–101.67	< 0.001
Service length (months)						
≤ 3 months	8	34	0.16	121.2	113.62–128.78	< 0.001
4∼6 months	8	66	0.005	108.78	98.69–118.87	< 0.001
7∼12 months	13	84	< 0.001	101.18	91.58–110.77	< 0.001
13∼24 months	6	56	0.05	103.47	93.05–113.90	< 0.001
Working department						
Oncology	3	51	0.13	100.82	85.84–115.81	< 0.001
Intensive care unit	6	0	0.82	98.31	93.73–102.88	< 0.001
Emergency	7	9	0.36	107.92	102.18–113.65	< 0.001
Operating room	5	0	0.76	98.01	87.47–108.56	< 0.001
Pediatrics	4	0	0.70	98.00	80.62–115.37	< 0.001
Obstetrics	2	0	0.63	94.07	64.96–123.17	< 0.001

### 3.4. Subgroup Analysis of the Current Status of Transition Shock Among New Nurses in China

Given that the Chinese healthcare system and the working environment of new nurses have changed considerably over the past decade and that previous studies have suggested that transition shock may differ by service length and department, these factors are likely to contribute to between‐study heterogeneity. Therefore, we conducted subgroup analyses by year of publication, service length, and working department (Table 2). Across the three time periods, heterogeneity within subgroups decreased to moderate or low levels (*I* ^2^ = 58% for 2015–2018; *I* ^2^ = 0% for 2019–2022 and 2023–2025), suggesting that time period partly explained the overall heterogeneity. For service length, heterogeneity was relatively low among nurses with ≤ 3 months of service (*I* ^2^ = 34%) but remained substantial among those with 4–6 months, 7–12 months, and 13–24 months of service (*I* ^2^ = 66%, 84% and 56%, respectively). With regard to working department, heterogeneity was low or absent in most subgroups (*I* ^2^ ranging from 0% to 9%), except for oncology (*I* ^2^ = 51%), indicating that clinical department may also be an important moderator. In terms of pooled means, transition shock was highest among Chinese new nurses with ≤ 3 months of service and those working in high‐intensity departments, such as emergency, oncology, and intensive care units, and relatively lower among those working in operating rooms, pediatrics, and obstetrics.

### 3.5. Results of the Meta‐Analysis on Influencing Factors of Transition Shock Among New Nurses in China

The same influencing elements that were referenced in at least two of the 30 papers that were included in this study were extracted, resulting in a total of 20 influencing factors. According to the results of the heterogeneity test, the heterogeneity of two influencing factors, namely, the registered residence and social support, was low (*I* ^2^ < 50%), and a fixed‐effects model was used for meta‐analysis. The remaining 18 influencing factors had high heterogeneity (*I* ^2^ > 50%), so the random effects model was used. A meta‐analysis of the findings revealed that the following factors influenced the impact of the transition of new nurses in China: female, nonlocal origin, college degree or below, disliking or feeling neutral toward their department, choosing the nursing profession due to nonpersonal interests, low professional identity, poor psychological resilience, infrequent feedback‐seeking behavior, service length ≤ 6 months, nonestablishment employment type, monthly income ≤ 5000 RMB, > 7 night shifts per month, lack of family support for nursing work, and low social support (all *p* < 0.05) (Tables 3 and 4).

**Table 3:** Meta‐analysis results of influencing factors for transition shock among nurses in China (continuous variable).

Influencing factors	Experimental group	Control group	Number of included studies	Heterogeneity		Effect model	Meta‐analysis result		
				*I* ^2^ (%)	*p*		SMD (95% CI)	*Z*	*p*
Gender	Male	Female	18	99	< 0.001	Random	−1.31 (−2.04, −0.58)	3.52	< 0.001
Age	≤ 25	> 25	13	95	< 0.001	Random	0.23 (−0.05, 0.51)	1.61	0.11
Employment type	Establishment	Non‐establishment	14	95	< 0.001	Random	−0.75 (−1.25, −0.26)	2.97	0.003
Education background	Junior college degree or below	Bachelor degree or above	20	98	< 0.001	Random	0.66 (0.35, 0.97)	4.13	< 0.001
Service length (months)	≤ 6 months	> 6 months	11	96	< 0.001	Random	0.80 (0.36, 1.24)	3.57	< 0.001
Place of origin	Non‐local	Local	12	99	< 0.001	Random	1.86 (1.02, 2.69)	4.34	< 0.001
Monthly income (CNY)	≤ 5000	> 5000	10	98	< 0.001	Random	0.45 (0.04, 0.86)	2.16	0.03
Only child status	Yes	No	14	95	< 0.001	Random	−0.01 (−0.29, 0.27)	0.04	0.97
Frequency of night shifts	≤ 7/month	> 7/month	6	98	< 0.001	Random	−0.85 (−1.57, −0.13)	2.320	0.02
Weekly working hours	≤ 40 h	> 40 h	3	92	< 0.001	Random	−0.49 (−1.00, 0.002)	1.9	0.06
Department liking	Like	Neutral/dislike	4	92	< 0.001	Random	−0.78 (−1.24, −0.32)	3.36	< 0.001
Registered residence	Urban	Rural	3	0	0.48	Fixed	−0.01 (−0.18, 0.15)	0.14	0.89
Reasons for choosing nursing profession	Personal interest	Other	3	93	< 0.001	Random	−0.60 (−1.16, −0.05)	2.12	0.03
Hospital level	Second level	Tertiary	4	99	< 0.001	Random	−0.57 (−1.69, 0.56)	0.99	0.32
Marital status	Unmarried	Married	13	91	< 0.001	Random	−0.13 (−0.40, 0.15)	0.90	0.37
Family support	Yes	No	6	73	0.002	Random	−0.38 (−0.69, −0.06)	2.32	0.02

**Table 4:** Meta‐analysis results of influencing factors for transition shock among nurses in China (related variables).

Influencing factors	Number of included studies	Heterogeneity		Effect model	Meta‐analysis result		
		*I* ^2^ (%)	*p*		Summary Fisher’s *Z* (95% CI)	*p*	Summary *r* (95% CI)
Professional identity	5	92	< 0.001	Random	−0.65 (−0.84, −0.46)	< 0.001	−0.57 (−0.68, −0.43)
Psychological resilience	4	99	< 0.001	Random	−0.59 (−0.64, −0.53)	< 0.001	−0.53 (−0.56, −0.49)
Feedback‐seeking behavior	5	99	< 0.001	Random	−1.00 (−1.79, −0.22)	0.01	−0.76 (−0.94, −0.22)
Social support	2	44	0.18	Fixed	−0.36 (−0.43, −0.29)	< 0.001	−0.35 (−0.41, −0.28)

### 3.6. Sensitivity Analysis

Sensitivity analyses using different effect models showed no significant changes in the status of transition shock or related influencing factors among new nurses in China, indicating the stability of the meta‐analysis findings.

### 3.7. Publication Bias

**Figure 3:**
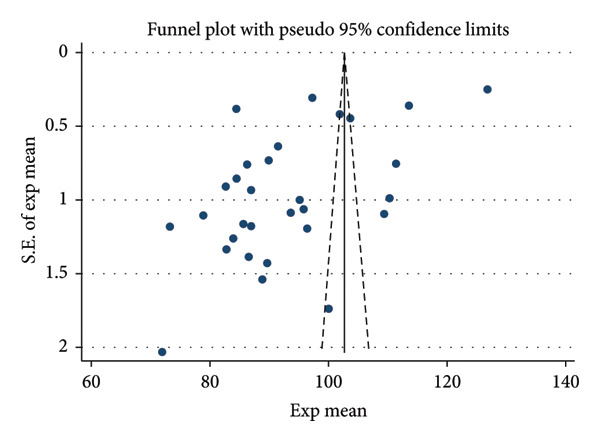
Funnel diagram of the literature on the current status of transition shock among new nurses in China.

The current status and the influencing factors included in ≥ 10 publications were subjected to Egger’s test for publication bias. The results showed that, with the exception of gender, Egger’s test produced a *p* value greater than 0.05, suggesting that the included studies had a relatively low likelihood of publication bias (Figure 3, Table 5).

**Table 5:** Results of Egger’s test for current status and influencing factors.

Influencing factors	Egger’s test	
	*t*	*p*
Current status	0.18	0.858
Gender	3.98	0.001
Employment type	−0.35	0.736
Education background	0.55	0.588
Service length (months)	−0.06	0.957
Place of origin	−1.37	0.202
Monthly income	−0.35	0.737

## 4. Discussion

The current status of transition shock and its influencing factors among new nurses in China were evaluated in this systematic study and meta‐analysis. After a thorough search of both local and foreign databases, 30 articles involving 12,459 Chinese new nurses were included. According to the meta‐analysis’ findings, the pooled transition shock score of Chinese new nurses was 93.08, with a score rate of 68.9%, indicating a moderate‐to‐high level of transition shock. Although direct numerical comparison is limited by differences in instruments, this moderate‐to‐high level is broadly consistent with international studies from the Philippines, Korea, and other countries, which also report substantial transition difficulties among newly graduated nurses and link higher transition shock to poorer job outcomes and patient outcomes [46, 47].

In the overall meta‐analysis, the heterogeneity of the pooled transition shock score was extremely high (*I* ^2^ = 99.8%), indicating marked differences in transition shock levels across settings and study populations. Subgroup analyses by publication year, service length, and working department helped to explain part of this variability, with time period and clinical environment in particular reducing heterogeneity to moderate or low levels in most strata. In contrast, heterogeneity across several service‐length categories remained considerable, implying that additional unmeasured organizational or individual factors are likely to influence the magnitude of transition shock.

The level of transition shock among new nurses in China varies by time dimension, service length, and department. The level of transition shock among new nurses in China shows a trend of gradual decrease in the time dimension, which is consistent with the “transition shock theory” proposed by Duchscher [8]. This theory posits that the pressure faced by new nurses in adapting to their profession will gradually diminish as the support and training systems are enhanced. China’s nursing education system has been getting better in recent years, particularly with regard to the practice‐oriented curriculum reforms that have advanced significantly [48]. These reforms have facilitated the alignment of nursing education with actual clinical demands, thereby laying a sturdy foundation for new nurses to gather hands‐on experience. Additionally, the nursing management mode has been optimized, and new nurses can receive one‐on‐one support and advice through the mentorship system that has been developed in recent years. According to Li et al. [49], the mentorship system can give new nurses the opportunity to get timely professional advice when they run into issues in their actual work, which will boost their confidence and capacity to handle work‐related challenges and hasten the transition from student to nurse.

According to Duchscher’s findings, transition shock among new nurses exhibited a nonlinear trend and was most acute 1–4 months after entering the profession [8]. According to subgroup studies, the highest level of transition shock was observed among Chinese new nurses who had been employed for ≤ 3 months. The intensity of transition shock among Chinese new nurses diminished progressively as their service length increased, aligning with the findings from prior longitudinal research [47, 50]. According to a study by Ouyang [51] of 9 new nurses in Hengyang City, Hunan Province, their transition shock was divided into three stages: knowledge and skill pressure during the preservice training period, negative emotions brought on by inexperience during the independent onboarding period, and interpersonal shock during the business familiarization phase. This dynamic transition shock, evolving over time, implies that nursing managers should implement segmented and stepwise training programs for new nurses, tailored to their needs and characteristics at different stages, to facilitate their scientific and smooth transition period. To lessen the transition shock of new nurses, Tian et al. [52], for instance, developed a nursing safety training content system based on the theory of transition shock and separated the new nurses into three stages over the course of a year, based on their psychological traits and educational requirements at each stage.

In China, there are significant differences in the shock faced by new nurses in different departments during the transition process. According to the study’s findings, new nurses experienced a far greater degree of transition shock in the emergency than in other departments. The high‐stress environment of unexpected life‐support procedures, the demanding demands for quick decision‐making skills, and the high‐pressure resuscitation rhythm of emergency care are likely to cause decision‐making anxiety and skill stress in new nurses [28]. Conversely, a different study [31] revealed that new nurses experienced the most transition shock in intensive care units. The unstable nature of the patient’s condition, the high‐intensity life‐support technical demands of the intensive care unit, and the demanding communication context of multidisciplinary collaboration may all contribute to this. These factors tend to cause technical anxiety and emotional exhaustion in new nurses [53], which makes transition adaptation challenging. Although new nurses in various departments have varying degrees of transition shock, it is alarming that new nurses in China typically experience significant levels of transition shock. To strengthen the evidence for reducing transition shock, future research should look more closely at the factors driving transition shock among new nurses in certain units. When creating training and support plans, nursing managers should also take into account the unique features of certain departments and use specialized management techniques.

The occurrence of transition shock for new nurses in China is mainly influenced by individual and external factors. The study’s findings indicate that gender, education background, and place of origin are important demographic factors that affect how new nurses in China adjust to their new roles. Today’s workplace is still subtly influenced by the traditional Chinese cultural gender division of labor model, which holds that “male dominates the outside and female dominates the inside.” Female new nurses experience a significantly higher level of transition shock than male new nurses because they are primarily in their prime reproductive years, must balance a demanding clinical workload with family role responsibilities [54], and may experience physical weakness due to specific physiological phases such as menstruation and pregnancy [32]. As a result, compared to male new nurses, female new nurses experienced noticeably higher levels of transition shock. Secondly, nonlocal origin nurses may face geographical adjustment challenges upon entering the profession and must swiftly familiarize themselves with the hospital environment, colleague relationships, and local linguistic norms. Being separated from friends and family also makes it harder to address their emotional needs and results in a lack of social support, which makes the transition shock even worse [20]. Furthermore, the curriculum for new nurses with college degree or below is centered on clinical nursing knowledge and skill operation [17], and research training is comparatively weak, which leads to limited career development opportunities and insufficient competitiveness in the declaration of scientific research topics and career advancement [55]. This is strongly tied to the contemporary nursing industry’s predilection for highly educated individuals. Hospitals typically prioritize academic credentials for promotion and research budget allocation, which exacerbates the marginalization of low‐education groups. Similar patterns have been reported in other countries, where lower resilience, weaker social support, and less favorable organizational conditions are also associated with higher transition shock, burnout, and turnover intention among newly graduated nurses, indicating that these are core, cross‐cultural determinants of early career adjustment. Nursing managers should take the following actions in light of the aforementioned factors: improve the care given to female new nurses during their unique physiological period, rationalize work arrangements, and lessen their physiological burden, while simultaneously eliminating gender bias, giving them equal opportunities for career development; increase the area culture’s direction and establish an emotional support system for nonlocal origin nurses to ease the stress of the transfer and aid in their integration into new settings; strengthen the continuous education system and put up research ability development courses for low‐education nurses to boost the competitiveness of low‐education nurses.

As the primary forces behind career development, career awareness and motivation have a big impact on new nurses’ professional adaption process. The study’s findings demonstrated that transition shock among new nurses in China was influenced by factors such as reasons for choosing the nursing profession, department liking, and professional identity. The “departmental post” management model is currently widely used in Chinese medical institutions, and new nurses have little room for independent choice. This can quickly result in long‐term occupational psychological depletion when their skills and personal career interests do not align with the nature of their work in the department [56]. For the noninterest‐driven group of new nurses, the conflict between personal professional aspirations and family expectations, coupled with the discrepancy between career‐oriented decisions and clinical practice realities, poses significant challenges. This makes their psychological stress during the transition phase even worse, especially when combined with the social cognitive bias against the nursing profession. Therefore, by conducting career planning lectures and establishing role models, nursing managers ought to strengthen the mentorship of new nurses’ professional identities, help them recognize the importance and value of nursing work, and foster their innate motivation. Concurrently, the implementation of a departmental rotation trial postsystem and the creation of a “two‐way fit” mechanism protect clinical personnel by respecting their individual career development goals and lessening the impact of transitions brought on by disparities in departmental preferences.

The findings of this study are in line with those of Meyer and Shatto’s [57] and Cao et al. [58], which consistently demonstrated that strong psychological resilience helps new nurses to keep a stable mental state and lessen their transition shock as they move from being students to nurses. When dealing with the effects of intricate interpersonal connections and challenging clinical work, nurses who possess strong psychological resilience are able to look at the bright side of things and take the initiative to take action to mitigate the negative effects of unfavorable events [44]. Furthermore, the results of this study demonstrated that new nurses’ feedback‐seeking conduct might reduce their degree of transition shock, which is in line with the findings of Dai et al. [59]. Feedback‐seeking activity is also essential for managing transition stress. In addition to helping new nurses adjust to their surroundings, feedback‐seeking behavior is a positive socialization technique that can encourage people to think creatively in order to achieve the right goals, boost their sense of professional worth and fulfillment, and lessen transition shock [60]. Therefore, when creating intervention programs, nursing managers should concentrate on building psychological capital and a feedback support system. They should also use clinical mentorship or narrative reflection workshops to assist new nurses in navigating the transition period.

The results of this study show that employment type, monthly income, and night shift frequency are work factors among the external factors affecting the transition shock of new nurses in China. China’s healthcare sector maintains a dual employment type system, categorizing nurses into establishment‐based and nonestablishment personnel based on their institutional affiliation status. As a result, nonestablishment nurses are frequently at a disadvantage when it comes to pay and treatment, opportunities for advancement, and access to training materials, among other things. A feeling of unfairness and estrangement at work can quickly result from this systemic disparity [61]. According to Yu and Li [62], over 86% of new nurses leaving China felt that their pay was insufficient to cover their personal expenses. The study’s findings also demonstrated that new nurses earning less than 5000 RMB per month experienced a greater degree of transition shock and that low‐income new nurses had to cope with the financial burden caused by vocational training and skill upgrading on top of meeting their basic living needs. Therefore, in order to create a strong material foundation for the transformation and growth of new nurses, healthcare organizations should set up a fair and appropriate compensation system in addition to offering fair and transparent career development and promotion chances. According to the study’s findings, new nurses who worked more than seven night shifts each month experienced a noticeably higher degree of transition shock. Due to the excessive workload and the Chinese healthcare system’s “three‐shift” schedule pattern, new nurses experience chronic disruptions in their circadian rhythms [63]. Furthermore, frequent night shifts cause physiological deterioration and sleep loss, but they also cause long‐term psychological stress because new nurses are not clinically experienced and must handle complex clinical problems on their own [64]. These employment‐related factors may be particularly salient in the Chinese health system, where dual employment structures, relatively low starting salaries and heavy night‐shift loads are more pronounced than in many high‐income countries. To lessen the impact of frequent night shifts on new nurses’ role transition, healthcare organizations should establish a flexible scheduling system, improve nighttime manpower allocation, lower the nighttime workload through smart nursing systems, and offer new nurses’ professional sleep interventions and psychological counseling.

The findings of this study demonstrated that low levels of social support and “unsupportive attitudes” regarding nursing held by family members were significant contributors to the transition shock experienced by new nurses in China. During the transition period, new nurses typically encounter issues such as inadequate assistance and tasks that exceed their capabilities, which hinder their capacity to become certified registered nurses [65]. Additionally, a strong support network can lessen the effects of the transition phase, enhance a new nurse’s capacity to handle demands at work and lower stress levels, and lessen the severity of transition shock [66]. In order to lessen the severity of transition shock and enable new nurses to successfully complete their move, nursing administrators and society at large should offer them professional support and psychological counseling.

**Figure 4:**
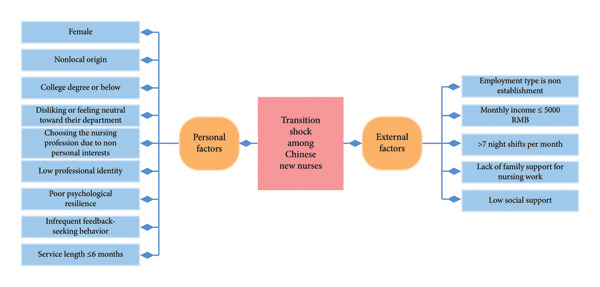
Influencing factors of transition shock among Chinese new nurses.

From a nursing management perspective, our findings show that transition shock among new nurses is shaped by clusters of personal and external factors rather than by any single variable. Personal resources such as resilience, professional identity, and active feedback‐seeking behavior can help new nurses cope with unfamiliar roles, whereas supportive organizational and family environments reduce the intensity of shock. Considering these elements together provides a useful basis for designing transition programs and retention strategies that simultaneously strengthen individual coping capacity and improve working conditions. These relationships are summarized in a simple conceptual framework (Figure 4), which highlights how personal and external factors jointly influence transition shock and related retention outcomes. Although our data come from China, these management implications are likely to be relevant to other health systems where newly graduated nurses face similar pressures during the transition from student to independent practitioner.

Through subgroup analyses of different time periods, lengths of service, and departments, this study further investigated the heterogeneity of the current status of transition shock among new nurses in China and identified several potential moderators, providing an evidence base for the development of tailored prevention and intervention strategies. Nevertheless, even after subgrouping, substantial residual heterogeneity remained in some strata, so the pooled estimates should be interpreted with caution and regarded mainly as an indication of the general range and direction of transition shock rather than as a precise single value. In addition, this study has several limitations: (i) A less thorough examination of transition shock among male new nurses may have resulted from the higher percentage of female new nurses among the 30 included studies. (ii) There is a higher chance of selection and publication bias because the literature considered in this study was cross‐sectional and lacked conventional randomized controlled trials. Therefore, future studies should pay attention to balancing the gender ratio and conduct more large‐sample, multicenter studies to further analyze the current status of transition shock among new nurses and related influencing factors.

## 5. Conclusion

Among Chinese new nurses, transition shock was prevalent overall and varied by time dimension, service length, and department. Gender, employment type, education background, service length, place of origin, monthly income, frequency of night shifts, department liking, reasons for choosing nursing profession, family support, professional identity, psychological resilience, feedback‐seeking behavior, and social support were all factors that influenced transition shock in new Chinese nurses. In order to improve the career satisfaction of new nurses and the quality of their care, nursing managers can use the findings of this meta‐analysis to better assess and identify the factors influencing the transition shock of new nurses in China and implement effective interventions to reduce the transition shock of new nurses as much as possible. While our meta‐analysis focused on China, the consistency of several key determinants with international studies suggests that strategies to strengthen resilience, social support, and fair employment conditions for new nurses may have relevance for other health systems facing nursing shortages.

## Conflicts of Interest

The authors declare no conflicts of interest.

## Author Contributions

Jiexuan Xu and Jianqin Huang contributed equally to this article as co‐first authors, with Jiexuan Xu listed as the first co‐first author and Jianqin Huang as the second co‐first author.

## Funding

No funds were received.

## Supporting Information

Supporting Tables S1 and S2 report the AHRQ item ratings and total scores for included studies (1–30), and Supporting Table S3 details the AHRQ 11‐item cross‐sectional/prevalence checklist and response options (Yes/No/Unclear).

## Supporting information


**Supporting Information** Additional supporting information can be found online in the Supporting Information section.

## Data Availability

The data that support the findings of this study are available from the corresponding author upon reasonable request.
