# Supplementary material for: Transition Shock Among Chinese New Nurses: A Systematic Review and Meta‐Analysis
Source: J Nurs Manag. 2026 Jan 14;2026:9306693. doi: 10.1155/jonm/9306693 (PMC12801397; doi:10.1155/jonm/9306693)
Supplement: Supplementary file 1 — Supporting Information Additional supporting information can be found online in the Supporting Information section. [file JONM-2026-9306693-s001.docx]

Supplementary Tables S1–S2 report the AHRQ item ratings and total scores for included studies (1–30), and Supplementary Table S3 details the AHRQ 11-item cross-sectional/prevalence checklist and response options (Yes/No/Unclear).

Table S1 AHRQ quality scores(studies 1–15)

| **Item** | **Liu and Gong, 2019** | **Ma et al., 2022** | **Liu et al., 2023** | **Tian et al., 2018** | **Chen et al., 2021** | **Zhang, 2024** | **Li and Wang, 2024** | **Duan, 2019** | **Ma, 2020** | **Huang and Li, 2022** | **Xue et al., 2015** | **Zhang, 2018** | **Song et al., 2021** | **Luo et al., 2024** | **Kong and Hu, 2022** |
| --- | --- | --- | --- | --- | --- | --- | --- | --- | --- | --- | --- | --- | --- | --- | --- |
| 1 | Yes | Yes | Yes | Yes | Yes | Yes | Yes | Yes | Yes | Yes | Yes | Yes | Yes | Yes | Yes |
| 2 | Yes | Yes | Yes | Yes | Yes | Yes | Yes | Yes | Yes | Yes | Yes | Yes | Yes | Yes | Yes |
| 3 | Yes | Yes | Yes | Yes | Yes | Yes | Yes | Yes | Yes | Yes | No | Yes | No | Yes | Yes |
| 4 | No | Yes | Yes | Yes | Yes | Yes | Yes | Yes | Yes | Yes | Yes | Yes | Yes | Yes | Yes |
| 5 | Unclear | Unclear | Unclear | Unclear | Unclear | Unclear | Unclear | Unclear | Unclear | Unclear | Unclear | Unclear | Unclear | Unclear | Unclear |
| 6 | Yes | Yes | Yes | Yes | Yes | Yes | Yes | Yes | Yes | Yes | Yes | Yes | Yes | Yes | Yes |
| 7 | No | Yes | No | No | Yes | No | No | Yes | Yes | No | Yes | No | No | No | No |
| 8 | Yes | Yes | Yes | Yes | Yes | Yes | Yes | Yes | Yes | Yes | Yes | Yes | Yes | Yes | Yes |
| 9 | Yes | No | Yes | No | No | Yes | No | Yes | Yes | No | No | Yes | No | No | No |
| 10 | Yes | Yes | Yes | Yes | Yes | Yes | Yes | Yes | Yes | Yes | No | Yes | Yes | Yes | Yes |
| 11 | Unclear | Unclear | Unclear | Unclear | Unclear | Unclear | Unclear | Unclear | Unclear | Unclear | Unclear | Unclear | Unclear | Unclear | Unclear |
| Scores | 7 | 8 | 8 | 7 | 8 | 8 | 7 | 9 | 9 | 7 | 6 | 8 | 6 | 7 | 7 |

Table S2 AHRQ quality scores(studies 16–30)

| **Item** | **Lin et al., 2023** | **Liu et al., 2016** | **Su and Su, 2019** | **Yao et al., 2023** | **Kang et al., 2023** | **Shi et al., 2022** | **Zheng et al., 2025** | **Huang et al., 2024** | **Chen et al., 2017** | **Zhu et al., 2024** | **Guan et al., 2021** | **Wu et al., 2022** | **Ma et al., 2022** | **Guo, 2019** | **Yu et al., 2018** |
| --- | --- | --- | --- | --- | --- | --- | --- | --- | --- | --- | --- | --- | --- | --- | --- |
| 1 | Yes | Yes | Yes | Yes | Yes | Yes | Yes | Yes | Yes | Yes | Yes | Yes | Yes | Yes | Yes |
| 2 | Yes | Yes | Yes | Yes | Yes | Yes | Yes | Yes | Yes | Yes | Yes | Yes | Yes | Yes | Yes |
| 3 | Yes | Yes | Yes | Yes | No | Yes | Yes | Yes | Yes | Yes | Yes | Yes | Yes | Yes | Yes |
| 4 | Yes | Yes | Yes | Yes | Yes | Yes | Yes | Yes | Yes | Yes | Yes | Yes | Yes | Yes | Yes |
| 5 | Unclear | Unclear | Unclear | Unclear | Unclear | Unclear | Unclear | Unclear | Unclear | Unclear | Unclear | Unclear | Unclear | Unclear | Unclear |
| 6 | Yes | Yes | No | Yes | Yes | Yes | Yes | Yes | Yes | Yes | Yes | Yes | Yes | Yes | Yes |
| 7 | No | No | No | No | No | Yes | No | No | No | No | Yes | No | Yes | Yes | Yes |
| 8 | Yes | No | Yes | Yes | Yes | Yes | Yes | Yes | Yes | Yes | Yes | Yes | Yes | Yes | Yes |
| 9 | Yes | Yes | Yes | Yes | No | No | No | Yes | No | No | No | No | Yes | Yes | Yes |
| 10 | Yes | Yes | Yes | Yes | Yes | Yes | Yes | Yes | Yes | Yes | Yes | No | Yes | Yes | No |
| 11 | Unclear | Unclear | Unclear | Unclear | Unclear | Unclear | Unclear | Unclear | Unclear | Unclear | Unclear | Unclear | Unclear | Unclear | Unclear |
| Scores | 8 | 7 | 6 | 8 | 6 | 8 | 7 | 8 | 7 | 7 | 8 | 6 | 9 | 9 | 8 |

Table S3 AHRQ quality scores

| **Item** | **Yes** | **No** | **Unclear** |
| --- | --- | --- | --- |
| 1) Define the source of information (survey, record review) |  |  |  |
| 2) List inclusion and exclusion criteria for exposed and unexposed subjects (cases and controls) or refer to previous publications |  |  |  |
| 3) Indicate time period used for identifying patients |  |  |  |
| 4) Indicate whether or not subjects were consecutive if not population-based |  |  |  |
| 5) Indicate if evaluators of subjective components of study were masked to other aspects of the status of the participants |  |  |  |
| 6) Describe any assessments undertaken for quality assurance purposes (e.g., test/retest of primary outcome measurements) |  |  |  |
| 7) Explain any patient exclusions from analysis |  |  |  |
| 8) Describe how confounding was assessed and/or controlled. |  |  |  |
| 9) If applicable, explain how missing data were handled in the analysis |  |  |  |
| 10) Summarize patient response rates and completeness of data collection |  |  |  |
| 11) Clarify what follow-up, if any, was expected and the percentage of patients for which incomplete data or follow-up was obtained |  |  |  |
